# Age-specific risk factors of severe pneumonia among pediatric patients hospitalized with community-acquired pneumonia

**DOI:** 10.1186/s13052-021-01042-3

**Published:** 2021-04-23

**Authors:** Lumin Chen, Chong Miao, Yanling Chen, Xian Han, Ziying Lin, Hong Ye, Chengyi Wang, Huijie Zhang, Jingjing Li, Qiuyu Tang, Yuan Dong, Meng Bai, Yibing Zhu, Guanghua Liu

**Affiliations:** 1 Department of Pediatrics, Fujian Maternity and Children’s Hospital, Affiliated Hospital of Fujian Medical University, Fuzhou, 350001 China; 2 Information Center, Fujian Maternity and Children’s Hospital, Affiliated Hospital of Fujian Medical University, Fuzhou, 350001 China; 3 Unimed Scientific, Inc., Wuxi, China

**Keywords:** Severe community-acquired pneumonia, Risk factors, Age, Pediatrics

## Abstract

**Background:**

Risk factors that predispose the development of severe community-acquired pneumonia (CAP) among pediatric CAP patients of different age ranges are yet to be identified.

**Methods:**

We retrospectively analyzed pediatric in-patients (< 6 years old) diagnosed with CAP in our hospital. We subdivided patients into four age groups (< 6 months, 6 months-1 year, 1–2 years, and 2–6 years). Their medical records, including demographic information, clinical features, laboratory findings, and chest radiographic reports, were reviewed and collected for further analysis. Univariate logistic regression analysis and stepwise regression analysis were applied to identify risk factors associated with severe CAP and ICU admission for overall patients and age-stratified subgroups.

**Results:**

A total of 20,174 cases were initially included. Among them, 3309 (16.40%) cases were identified as severe CAP, and 2824 (14.00%) cases required ICU admission. Potential risk factors for severe CAP and ICU admission identified by univariate analysis included younger age, rural residency, premature birth, low birth weight (LBW), formula feeding, congenital heart disease (CHD), history of pneumonia or neonatal jaundice, patients with other health issues, certain symptoms (manifesting wheezing, dyspnea, cyanosis, but have no cough or fever), abnormal laboratory findings (abnormal levels of white blood cells, albumin, and C-reactive protein and RSV infection), and chest X-ray (odds ratio [OR] > 1 for all). CHD, low albumin, proteinuria, abnormal chest x-ray were independent risks factors across different age groups, whereas birth or feeding history, history of pneumonia, cyanosis or dyspnea on admission, and RSV infection were independent risk factors for only younger kids (< 1 year), and wheezing was an independent risk factor only for older children (2–5 years old).

**Conclusions:**

Risk factors predicting disease severity among children hospitalized with CAP vary with age. Risk factor stratification of pediatric CAP based on age-specific risk factors can better guide clinical practice.

**Trial registration:**

This study has been registered in China, with the registration number being ChiCTR2000033019.

## Introduction

Community-acquired pneumonia (CAP) remains the most common reason for pediatric clinical visits and the major cause of pediatric mortality, posing a significant burden to the health system worldwide [1, 2]. According to a report by the World Health Organization (WHO), CAP killed 808,694 children in 2017, accounting for 15% of all deaths of children under 5 years old [3]. The status quo is even worse for developing countries like China, where more than 20 million new pediatric CAP cases are reported annually [4].

CAP is a pulmonary infectious disease acquired outside of the hospital, with viruses and bacteria as the most common pathogens [5]. Its severity varies dramatically from one person to another. Mild cases could recover swiftly even without specific treatment, whereas severe ones might end up with a dismal outcome even with intensive care [6]. In the era of precision medicine, we do not want to overtreat the mild cases or risk missing out on the severe disease, which might behave exactly like a mild disease at an early stage and early initiation of intensive treatment is critical in controlling its progression. Even with the advancement of modern medicine, there is no substantial improvement in the management strategy and treatment outcome of pediatric CAP, which is significantly ascribed to the inability to accurately predict disease severity and administer early intensive treatment or prophylactic therapies to high-risk cases [7].

Previous studies had worked on the identification of risk factors associated with severe CAP and thus facilitated the risk stratification of CAP patients [8–13]. The major drawback of the previous research is the negligence of the heterogeneity among pediatric patients of different age ranges. Children are a specific population that undergoes rapid growth and biological development, with different age groups harboring different physiological features and illness susceptibility. Given the major causes of CAP differ with age among pediatric patients [14, 15], we assume the risk factors that predispose pediatric CAP patients to the development of the severe disease may also vary according to age stratification. Utilizing the abundant in-patients data of pediatric CAP patients hospitalized in our center between April 2012 and September 2019, we systematically analyzed the risk factors associated with the development of severe CAP or ICU admission among all the pediatric CAP patients younger than 6 years. Specifically, age-specific risk factors were evaluated for subgroup patients of different age ranges (1–6 months, 6 months – 1 year, 1 year – 2 years, and 2 years − 6 years). Our findings add to the knowledge of risk stratification for CAP and provide evidence on age-specific risk factors in predicting severe CAP, which could better guide pediatricians in decision-making clinical practice.

## Patients and methods

### Study subjects

This retrospective study involved pediatric patients diagnosed with CAP and hospitalized in Fujian Maternity and Child Health Hospital between April 2012 and September 2019. Only the cases who qualified the following inclusion criteria were included: 1) pediatric patients between 1 month and 6 years old; 2) patients with discharge diagnosis ICD-10 codes containing J09-J18 (influenza and pneumonia) and J20-J22 (other acute lower respiratory infections); 3) patients hospitalized with CAP as the primary diagnosis. Cases with any of the following conditions were excluded from further analysis: 1) patients who were re-hospitalized within 1 week; 2) patients for whom clinical information was not available.

We defined patients with severe CAP based on the diagnostic criteria proposed in the clinical practice guidelines by the Pediatric Infectious Diseases Society and the Infectious Diseases Society of America [16]. Patients were classified into severe CAP cohort and non-severe CAP cohort for further analysis. We also categorized patients into ICU cohort and non-ICU cohort based on the ICU admission. According to the guidelines [17] for pediatric age groups, pediatric patients were suggested to be classified as: Preterm (birth < 37 weeks postmenstrual age [PMA]), Term neonatal (birth-27 days), Infants (28 days-12 months), toddler (13 months-2 years), early childhood (2–5 years), middle childhood (6–11 years), early adolescence (12–18 years), late adolescence (19–21 years). The majority of hospitalized patients in our hospital were below 6 years. As the condition of newborns is very much different from other age groups, we restricted our study population to patients aged between 1 month to 6 year old. We classified the patients into four age groups for further subgroup analysis: patients aged between 1 and 6 months, patients aged between 6 months and 1 year, patients aged between 1 year and 2 years, and patients aged between 2 years and 6 years. We subclassified the infants into two age groups (1–6 months and 6–12 months) because of the large sample size of infant patients and also because we want to analyze the difference between infant stage and late infant stage in detail.

### Data collection

Hospital records of all pediatric patients were retrospectively screened through the electronic information system of our hospital. We identified cases meeting the upper mentioned criteria through the algorithm designed by the programmer. Detailed medical records of the eligible cases were reviewed by trained investigators to further confirm their qualification for final inclusion, as well as to confirm the classification of severe CAP cohort and non-CAP cohort. Medical information collected for subsequent analysis included: 1) epidemiological data pertaining to age, gender, resident area, birth history (birth weight, birth terms), feeding pattern (breastfeeding, formula feeding, or mixture), 2) medical history (history of pneumonia or neonatal jaundice), and concomitant diseases (congenital heart disease); 3) laboratory findings (blood routine, biochemical routine, inflammatory markers, biomarkers of liver function and kidney function, pathogen detection of the respiratory syncytial virus [RSV]); 4) chest radiographic reports; 5) clinical manifestations (the presence of cough or wheezing); 6) clinical management (ICU admission or not). The blood routine test, biochemical routine test, and chest radiography were carried out right after admission. Description of symptoms was derived from the history of present history, which was obtained at admission. Radiographic results were classified into two categories: normal radiographs and abnormal radiographs, which showed signs of pneumonia with or without other abnormalities like emphysema, consolidation, atelectasis, pneumothorax, and hydrothorax. RSV detection was carried out by antigen detection, with the elevation of RSV-specific IgM defined as RSV positive. Continuous variables like white blood cell (WBC) count, neutrophil counts, lymphocyte counts, serum sodium level, aspartate aminotransferase (AST), alanine aminotransferase (ALT), total bilirubin (TBIL), serum creatine, C-reactive protein (CRP), Erythrocyte Sedimentation Rate (ESR), and serum albumin (ALB) levels were transferred in categorical variables based on the thresholds that divided the value into normal and abnormal ones. Normal range for WBC count, neutrophil counts, lymphocyte counts were specified according to different age groups: WBC count, 5.0–15*10^9/L for 1 m–6 m, 6.0–17.5*10^9/L for 6 m–12 m, 1.0–8.5*10^9/L for 1y-6y; neutrophil count 1.0–5*10^9/L for 1 m–6 m, 1.0–8.5*10^9/L for 6 m-6y; lymphocytes count 4.0–10*10^9/L for 1 m–6 m, 4.0–12*10^9/L for 6 m–12 m, 1.5–9.5*10^9/L for 1y-6y. Any value that falls beyond the normal range divided by age will be classified as abnormal. We defined low birth weight (LBW) as birth weight lower than 2.5Kg, and fever as a temperature higher than 37.5 °C.

### Statistical analysis

The SAS 9.4 for windows (SAS Institute Inc., Cary, NC, USA) was used to carry out the statistical analysis. Continuous variables were presented as mean ± standard deviation (SD), and inter-group differences were assessed using the independent *t*-test. Inter-group differences with respect to categorical variables were assessed using the χ2 test, CMH-χ2 test, or Fisher’s exact test where appropriate. Logistic regression univariate analysis was conducted to explore potential risk factors for developing severe CAP or requiring ICU admission among children hospitalized with CAP. Factors demonstrating significant relevance in univariate analysis were further evaluated with stepwise regression analysis to identify independent risk factors associated with severe CAP or ICU admission. All the analyses were performed on the overall included patients, as well as for subgroups stratified by age. Statistical tests were interpreted at a two-sided significance level of 0.05.

## Results

### Clinical features of the study population

A total of 20,174 cases were qualified for the final inclusion in the study, among which, 3309 (16.40%) cases met the diagnostic criteria of severe CAP, and 2824 (14.00%) required ICU admission during the hospitalization (Table 1). As for age distribution, there were 9291 (46.05%) cases under the age of 6 months, 3519 (17.44%) cases aged between 6 months and 1 year, 3309 (16.40%) cases aged between 1 year and 2 years, and 4055 (20.10%) cases aged between 2 years and 6 years (Table 1). There were more male patients (65.26%) among the overall pediatric patients hospitalized with CAP, as well as among patients of different age groups. Among all the enrolled cases, the most frequently observed symptoms at admission were cough (88.7%), fever (43.46%), and wheezing (14.68%) (Table 1). Breathless and cyanosis were much less frequent, accounting for 0.49 and 1.74%, respectively (Table 1). Signs of pneumonia and other abnormalities, such as consolidation, hydrothorax, and pneumothorax, were observed on chest radiography in 73.34% of the overall study cases, while the remaining 27.66% of patients had totally normal chest X-ray at admission. Of all the pediatric patients included, 2729 (13.53%) had preexisting congenital heart disease (CHD) as a comorbidity, and 2757 (13.67%) had a history of pneumonia (Table 1). A total of 10,434 cases developed complications during hospitalization, accounting for 51.72% of the enrolled subjects. Among patients with relevant data available, 14.83% of the patients required mechanical ventilation, the percentage of which is similar to that of ICU admission and severe CAP (Table 1).

**Table 1:** Clinical characteristics of pediatric CAP by age

	Total ***N*** = 20,174	< 6 m ***N*** = 9291	6 m-1y ***N*** = 3519	1y-2y ***N*** = 3309	2y-6y ***N*** = 4055	***P***
Gender (male) n(%)	13,165 (65.26)	6267 (67.45)	2458 (69.87)	2145 (64.82)	2295 (56.61)	< 0.0001
Symptoms on admission						
Cough	17,907 (88.76)	8069 (86.85)	3199 (90.91)	2940 (88.85)	3699 (91.22)	< 0.0001
Fever	8767 (43.46)	1668 (17.95)	1850 (52.57)	2211 (66.82)	3038 (74.92)	< 0.0001
Wheezing	2962 (14.68)	1352 (14.55)	825 (23.44)	469 (14.17)	316 (7.79)	< 0.0001
Breathless	99 (0.49)	69 (0.74)	13 (0.37)	11 (0.33)	6 (0.15)	< 0.0001
Cyanosis	352 (1.74)	308 (3.32)	22 (0.63)	18 (0.54)	4 (0.10)	< 0.0001
X-ray Manifestation						
Normal	3769 (27.66)	1483 (23.34)	758 (32.84)	627 (29.10)	901 (32.05)	< 0.0001
Pneumonia	8939 (65.59)	4401 (69.26)	1421 (61.57)	1413 (65.57)	1704 (60.62)	
Others	920 (6.75)	470 (7.40)	129 (5.59)	115 (5.34)	206 (7.33)	
CHD n(%)	2729 (13.53)	2172 (23.38)	320 (9.09)	152 (4.59)	85 (2.10)	< 0.0001
History of pneumonia n(%)	2757 (13.67)	947 (10.19)	634 (18.02)	539 (16.29)	637 (15.71)	< 0.0001
Severe pneumonia n(%)	3309 (16.40)	2187 (23.54)	377 (10.71)	353 (10.67)	392 (9.67)	< 0.0001
Had complications n(%)	10,434 (51.72)	4962 (53.41)	1839 (52.26)	1724 (52.10)	1909 (47.08)	< 0.0001
Treatment						
ICU admission	2824 (14.00)	1949 (20.98)	319 (9.07)	282 (8.52)	274 (6.76)	< 0.0001
Mechanical ventilation	378 (14.83)	229 (13.05)	47 (16.97)	42 (16.15)	60 (23.35)	< 0.0001

*CAP* Community-acquired pneumonia, *CHD* Congenital heart disease, *ICU* Intensive care unit

**Fig. 1:**
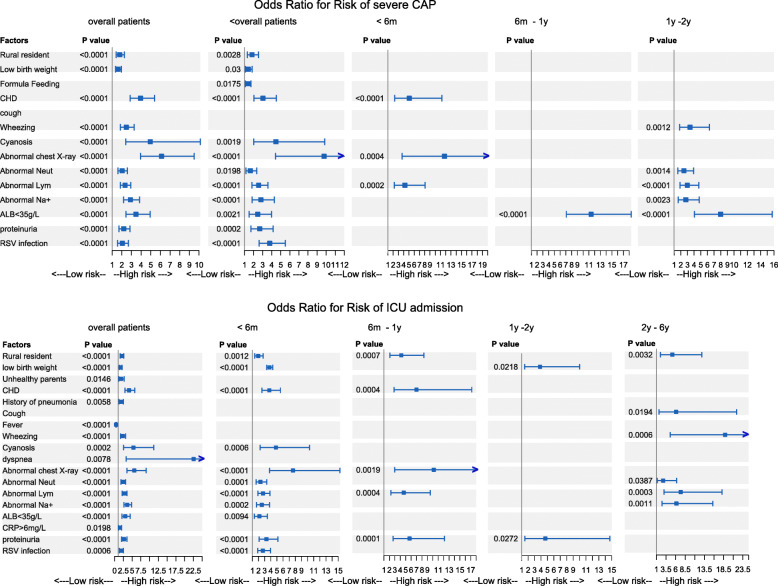
Multivariate analysis for Risk factors of severe CAP (**a**) or ICU admission (**b**) among overall patients and patients stratified by ages

We also detailly characterized the patients of different age groups in terms of symptoms on admission, chest radiographic findings, history of pneumonia, comorbidity of CHD, clinical management, etc. (Table 1). All these parameters varied with age. For example, fever as a symptom on admission was less common for infant patients younger than 6 months but was more commonly observed as the age increased. CHD as a comorbidity was much commonly observed among the youngest patients (< 6 months) hospitalized with CAP, yet the prevalence dropped significantly as the age increased. As for patients with a history of pneumonia, the percentage was lowest among patients younger than 6 months (10.19%) but the highest among patients aged between 6 months and 1 year (18.02%). The youngest CAP patients had the highest risk of developing severe CAP (23.54%) and requiring ICU admission (20.98%), whose incidence were all significantly decreased with age. Interestingly enough, mechanical ventilation was administrated more often to the older patients (2–6 years old) than the younger age groups.

### Potential risk factors for severe CAP identified by univariate analysis

In the present study, we defined severe CAP based on the diagnostic criteria derived from the clinical guidelines. ICU referral, as a simple and objective index, was also applied as an alternative measure of severe CAP. Univariate analysis was carried out to identify potential risk factors associated with severe CAP of ICU admission among all the enrolled cases (Tables 2 and 3). As for demographic features, younger ages and rural residency were associated with a higher risk of severe CAP or ICU admission (*P* <  0.0001), whereas the gender did not convey predictive value on disease severity (*P* > 0.05). Birth history, such as premature birth and low birth weight (LBW, < 2.5 Kg), was a significant predictor of severe CAP or ICU admission (*P* <  0.0001). The feeding method might also play a role in altering the risk for severe CAP among children, with breastfeeding acting as a protective factor, whereas formula feeding was associated with increased risk. When it comes to family history, children with at least one patient in an unhealthy status demonstrated a higher risk of developing severe CAP or requiring ICU referral. As expected, medical history like comorbidity with CHD and past history of pneumonia or neonatal jaundice were significant predictors of severe CAP or ICU admission (*P* <  0.0001). We further evaluated the correlation between clinical manifestations on admission and the risk of developing severe CAP during hospitalization. Unexpectedly, although cough and fever are the most commonly observed symptoms in patients with CAP, they were associated with a reduced risk of severe CAP or ICU admission (*P* <  0.0001). That is, patients without cough or fever had a higher tendency of developing severe CAP and requiring ICU referral. On the contrary, wheezing, dyspnea, and cyanosis were significant predictors of severe CAP or ICU admission (*P* <  0.0001). As for radiographic and laboratory findings on admission, abnormal chest X-ray (with signs of pneumonia or other abnormalities), abnormal WBCs (WBC count < 5.0*10^9/L or > 12*10^9/L), hypoalbuminemia (ALB < 35 g/L), elevated CRP (CRP > 6 mg/L), proteinuria, and positive on RSV detection were significant predictors of increased risk of severe CAP or ICU admission (*P* <  0.01).

**Table 2:** Univariate analysis for risk factors of severe CAP

	Severe CAP	Non-severe CAP	OR(95% CI)	***P***
Age n(%)				
< 6 m	2187 (66.09)	7104 (42.12)	0.67 [0.64–0.69]	< 0.0001
6 m-1y	377 (11.39)	3142 (18.63)		
1y-2y	353 (10.67)	2956 (17.53)		
2y-6y	392 (11.85)	3663 (21.72)		
Gender (male) n(%)	2192 (66.24)	10,973 (65.06)	1.05 [0.97–1.14]	0.1925
Rural resident n(%)	1638 (49.67)	5454 (32.50)	2.05 [1.90–2.21]	< 0.0001
Premature birth n(%)	749 (23.55)	1389 (8.34)	3.38 [3.07–3.73]	< 0.0001
LBW n(%)	744 (23.02)	1254 (7.58)	3.65 [3.30–4.03]	< 0.0001
Feeding				
Breastfeeding	1307 (40.25)	8811 (52.92)	1.42 [1.36–1.49]	< 0.0001
Mixture	1080 (33.26)	4957 (29.77)		
Formula	860 (26.49)	2881 (17.30)		
Unhealthy parents	383 (11.77)	1207 (7.23)	1.71 [1.51–1.93]	< 0.0001
CHD	1222 (36.93)	1507 (8.94)	5.97 [5.46–6.52]	< 0.0001
History of pneumonia	785 (23.72)	2000 (11.86)	2.31 [2.11–2.54]	< 0.0001
History of jaundice	231 (6.98)	420 (2.49)	2.94 [2.49–3.47]	< 0.0001
Symptoms at admission n(%)				
Cough	2574 (77.79)	15,333 (90.92)	0.35 [0.32–0.39]	< 0.0001
Fever	1123 (33.94)	7644 (45.32)	0.62 [0.57–0.67]	< 0.0001
Wheezing	577 (17.44)	2385 (14.14)	1.28 [1.16–1.42]	< 0.0001
Dyspnea	735 (22.21)	1532 (9.08)	59.58 [28.89–122.87]	< 0.0001
Cyanosis	278 (8.40)	74 (0.44)	20.81 [16.06–26.97]	< 0.0001
Abnormal chest X-ray	535 (16.17)	385 (2.28)	8.26 [7.20–9.47]	< 0.0001
Abnormal results n(%)				
WBC	1629 (88.68)	7435 (80.54)	1.89 [1.62–2.21]	< 0.0001
Neutrophils	880 (49.03)	2291 (24.95)	2.89 [2.61–3.21]	< 0.0001
Lymphocytes	1028 (57.27)	2634 (28.68)	3.33 [3.00–3.70]	< 0.0001
Blood serum	337 (31.44)	397 (10.75)	4.27 [3.78–4.81]	< 0.0001
CRP	641 (35.45)	2390 (26.89)	1.49 [1.34–1.66]	< 0.0001
ESR	202 (75.09%)	1201 (82.20%)	0.65 [0.48–0.89]	0.0062
ALB	199 (10.87)	270 (2.79)	8.70 [7.53–10.05]	< 0.0001
ALT	110 (10.24)	208 (5.60)	2.97 [2.45–3.60]	< 0.0001
AST	140 (13.00)	256 (6.89)	3.61 [3.03–4.30]	< 0.0001
TBIL	384 (35.75)	951 (25.63)	2.69 [2.37–3.06]	< 0.0001
SCR	26 (2.43)	87 (2.36)	1.88 [1.34–2.63]	0.8943
Proteinuria	511 (31.76)	1036 (13.74)	2.92 [2.58–3.31]	< 0.0001
RSV	212 (26.27)	267 (18.94)	1.53 [1.24–1.87]	< 0.0001

Definition of abnormal laboratory results: WBC count < 5.0*10^9/L or > 15*10^9/L for 1 m–6 m, < 6.0*10^9/L or > 17.5*10^9/L for 6 m–12 m, < 1.0*10^9/L or > 8.5*10^9/L for 1y-6y; neutrophil count < 1.0*10^9/L or > 5*10^9/L for 1 m–6 m, < 1.0*10^9/L or > 8.5*10^9/L for 6 m-6y; lymphocytes count < 4.0*10^9/L or > 10*10^9/L for 1 m–6 m, < 4.0*10^9/L or > 12*10^9/L for 6 m–12 m, < 1.5*10^9/L or > 9.5*10^9/L for 1y-6y; blood serum, < 135 or 145 mmol/L; CRP > 6 mg/L; ESR > 10 mm/h; ALB< 35 g/L; ALT > 100 U/L; AST > 100 U/L; TBIL>17umol/L

*CAP* Community-acquired pneumonia, *ICU* Intensive care unit, *LBW* Low birth weight (birth weight < 2.5Kg); Unhealthy parents, at least one parent has health condition, *CHD* Congenital heart disease, *WBC* White blood cell, *Neu* Neutrophil count, *Lym* Lymphocyte count, *Na* Serum sodium, *CRP* C-reactive protein, *ESR* Erythrocyte sedimentation rate, *ALB* Albumin, *AST* Aspartate aminotransferase, *ALT* Alanine aminotransferase (ALT), *TBIL* Total bilirubin (TBIL), *SCR* Serum creatine, *RSV* Respiratory syncytial virus

**Table 3:** Univariate analysis for Risk factors of ICU admission

	ICU Admission	No ICU Admission	OR(95% CI)	***P***
Age n(%)				
< 6 m	1949 (69.02)	7342 (42.32)	0.62 [0.59–0.64]	< 0.0001
6 m-1y	319 (11.30)	3200 (18.44)		
1y-2y	282 (9.99)	3027 (17.45)		
2y-6y	274 (9.70)	3781 (21.79)		
Gender (male) n(%)	1878 (66.50)	11,287 (65.05)	1.07 [0.98–1.16]	0.1343
Rural resident n(%)	1467 (51.98)	5625 (32.59)	2.24 [2.07–2.43]	< 0.0001
Premature birth n(%)	658 (24.38)	1480 (8.64)	3.41 [3.08–3.78]	< 0.0001
LBW n(%)	659 (23.98)	1339 (7.86)	3.70 [3.33–4.10]	< 0.0001
Unhealthy parents	340 (12.26)	1250 (7.28)	1.78 [1.57–2.02]	< 0.0001
Feeding				
Breastfeeding	1083 (39.22)	9035 (52.73)	1.45 [1.38–1.52]	< 0.0001
Mixture	934 (33.83)	5103 (29.78)		
Formula	744 (26.95)	2997 (17.49)		
CHD	1095 (38.77)	1634 (9.42)	6.09 [5.56–6.67]	< 0.0001
History of pneumonia	673 (23.83)	2112 (12.17)	2.26 [2.05–2.49]	< 0.0001
History of jaundice	207 (7.33)	444 (2.56)	3.01 [2.54–3.57]	< 0.0001
Symptoms at admission n(%)				
Cough	2145 (75.96)	15,762 (90.85)	0.32 [0.29–0.35]	< 0.0001
Fever	885 (31.34)	7882 (45.43)	0.55 [0.50–0.60]	< 0.0001
Wheezing	483 (17.10)	2479 (14.29)	1.24 [1.11–1.38]	< 0.0001
Dyspnea	679 (24.04)	1588 (9.15)	28.38 [17.01–47.36]	< 0.0001
Cyanosis	264 (9.35)	88 (0.51)	20.23 [15.84–25.84]	< 0.0001
Abnormal chest X-ray	461 (16.32)	459 (2.65)	8.26 [7.20–9.47]	< 0.0001
Abnormal results n(%)				
WBC	1318 (89.23)	7746 (80.76)	1.89 [1.62–2.21]	< 0.0001
Neutrophils	762 (53.14)	2409 (25.24)	3.36 [3.00–3.76]	< 0.0001
Lymphocytes	881 (61.44)	2781 (29.14)	3.87 [3.45–4.35]	< 0.0001
Blood serum	487 (33.94)	990 (9.96)	4.64 [4.09–5.28]	< 0.0001
CRP	542 (35.33)	2489 (27.17)	1.49 [1.34–1.66]	< 0.0001
ESR	131 (70.43%)	1272 (82.38%)	0.51 [0.36–0.72]	0.0001
ALB	173 (11.70)	296 (2.96)	9.14 [7.89–10.58]	< 0.0001
ALT	149 (10.37)	352 (3.53)	3.16 [2.59–3.86]	< 0.0001
AST	189 (13.13)	393 (3.94)	3.68 [3.07–4.43]	< 0.0001
TBIL	372 (25.89)	1047 (10.51)	2.98 [2.60–3.40]	< 0.0001
SCR	44 (3.07%)	139 (1.40%)	2.23 [1.58–3.14]	< 0.0001
Proteinuria	442 (33.01)	1105 (14.15)	2.92 [2.58–3.31]	< 0.0001
RSV	160 (25.56)	319 (20.05)	1.53 [1.24–1.87]	0.0046

Definition of abnormal laboratory results: WBC count < 5.0*10^9/L or > 15*10^9/L for 1 m–6 m, < 6.0*10^9/L or > 17.5*10^9/L for 6 m–12 m, < 1.0*10^9/L or > 8.5*10^9/L for 1y-6y; neutrophil count < 1.0*10^9/L or > 5*10^9/L for 1 m–6 m, < 1.0*10^9/L or > 8.5*10^9/L for 6 m-6y; lymphocytes count < 4.0*10^9/L or > 10*10^9/L for 1 m–6 m, < 4.0*10^9/L or > 12*10^9/L for 6 m–12 m, < 1.5*10^9/L or > 9.5*10^9/L for 1y-6y; blood serum, < 135 or 145 mmol/L; CRP > 6 mg/L; ESR > 10 mm/h; ALB< 35 g/L; ALT > 100 U/L; AST > 100 U/L; TBIL>17umol/L

*CAP* Community-acquired pneumonia, *ICU* Intensive care unit, *LBW* Low birth weight (birth weight < 2.5Kg); Unhealthy parents, at least one parent has health condition, *CHD* Congenital heart disease, *WBC* White blood cell, *Neu* Neutrophil count, *Lym* Lymphocyte count, *Na* Serum sodium, *CRP* C-reactive protein, *ESR* Erythrocyte sedimentation rate, *ALB* Albumin, *AST* Aspartate aminotransferase, *ALT* Alanine aminotransferase (ALT), *TBIL* Total bilirubin (TBIL), *SCR* Serum creatine, *RSV* Respiratory syncytial virus

To evaluate whether pediatric patients of different age range share the same risk factors for developing severe CAP or requiring ICU admission, univariate analysis was carried out in the subgroup patients stratified by age (Tables 4 and 5). Most of the risk factors described above conveyed the same predictive significance across patients of different groups. They included rural residency, CHD, history of pneumonia, cough, cyanosis, dyspnea, abnormal chest X-ray, hypoalbuminemia, elevated CRP, and proteinuria (*P* <  0.05). Yet, the predictive value of quite a few other factors varied with age. Some behaved as a significant predictor only among younger patients, while others demonstrated significant relevance only in older children. For example, premature birth, LBW, and patients’ health status significantly predicted an increased risk of severe CAP or ICU admission among children younger than 2 years old but were less significant or not significant among those aged between 2 years and 6 years. Similarly, the history of neonatal jaundice was associated with a significantly increased risk of severe CAP among the younger patients (< 1 year) but not among the older ones (1 year – 6 years). A similar trend was also observed for laboratory findings such as abnormal WBC, which was a significant predictor of severe CAP or ICU admission among patients younger than 1 year, and RSV infection, which was only associated with increased risk of severe CAP among patients born within 6 months. On the contrary, wheezing on admission was associated with increased risk of severe CAP or ICU admission only among children older than 1 year but conveyed no predictive value among the younger ones. Unexpectedly, gender was irrelevant to disease severity in most age groups except pediatric patients aged between 1 year and 2 years, where the male gender was significant associated with reduced risk of developing severe CAP or requiring ICU admission.

**Table 4:** Univariate analysis for Risk factors of severe CAP among patients stratified by ages

Variable	< 6 m		6 m-1y		1y-2y		2y-6y	
	OR(95% CI)	***P***	OR(95% CI)	***P***	OR(95% CI)	***P***	OR(95% CI)	***P***
Gender (male)	1.11 [1.00–1.23]	0.0547	0.82 [0.66–1.03]	0.0891	0.80 [0.63–1.00]	0.0476	0.97 [0.78–1.19]	0.7588
Rural resident	1.86 [1.69–2.05]	< 0.0001	1.55 [1.25–1.93]	< 0.0001	1.90 [1.52–2.37]	< 0.0001	2.44 [1.96–3.03]	< 0.0001
Premature birth	3.56 [3.15–4.03]	< 0.0001	2.73 [2.06–3.63]	< 0.0001	2.21 [1.58–3.09]	< 0.0001	1.48 [1.02–2.16]	0.0403
LBW	4.00 [3.52–4.54]	< 0.0001	3.90 [2.99–5.10]	< 0.0001	1.97 [1.40–2.78]	< 0.0001	1.30 [0.87–1.95]	0.1961
Unhealthy parents	1.65 [1.41–1.92]	< 0.0001	1.74 [1.22–2.47]	0.0023	1.63 [1.13–2.36]	0.0094	1.41 [0.97–2.05]	0.0683
Feeding	1.44 [1.35–1.53]	< 0.0001	1.41 [1.24–1.61]	< 0.0001	1.13 [0.98–1.30]	0.0967	1.11 [0.97–1.28]	0.1360
CHD	4.69 [4.22–5.21]	< 0.0001	5.75 [4.42–7.48]	< 0.0001	5.62 [3.96–7.98]	< 0.0001	3.62 [2.22–5.92]	< 0.0001
History of pneumonia	2.57 [2.27–2.91]	< 0.0001	2.76 [2.18–3.51]	< 0.0001	2.11 [1.60–2.77]	< 0.0001	1.55 [1.16–2.06]	0.0027
History of jaundice	2.24 [1.86–2.70]	< 0.0001	3.18 [1.93–5.25]	< 0.0001	1.91 [0.83–4.37]	0.1258	1.68 [0.64–4.37]	0.2897
Cough	0.36 [0.32–0.41]	< 0.0001	0.27 [0.21–0.36]	< 0.0001	0.52 [0.39–0.70]	< 0.0001	0.38 [0.29–0.51]	< 0.0001
Fever	1.09 [0.96–1.23]	0.1944	1.05 [0.84–1.30]	0.678	0.89 [0.71–1.13]	0.347	0.82 [0.65–1.03]	0.0938
Wheezing	1.01 [0.88–1.16]	0.903	1.06 [0.83–1.36]	0.6419	2.33 [1.79–3.03]	< 0.0001	3.36 [2.53–4.46]	< 0.0001
Cyanosis	13.01 [9.88–17.13]	< 0.0001	55.49 [16.35–188.37]	< 0.0001	43.68 [12.58–151.63]	< 0.0001	28.23 [2.93–272.06]	0.0039
Dyspnea	35.08 [15.16–81.15]	< 0.0001	103.08 [13.39–793.81]	< 0.0001	0.52 [0.00-I]	0.9651	47.16 [5.51–403.78]	0.0004
Abnormal chest X-ray	7.70 [6.31–9.40]	< 0.0001	15.81 [10.90–22.94]	< 0.0001	10.39 [7.07–15.27]	< 0.0001	7.37 [5.45–9.98]	< 0.0001
Abnormal WBC	2.43 [1.98–2.98]	< 0.0001	2.01 [1.32–3.05]	0.2253	1.29 [0.85–1.95]	0.2253	1.27 [0.87–1.86]	0.2155
Abnormal Neu	2.48 [2.16–2.85]	< 0.0001	2.99 [2.21–4.06]	< 0.0001	3.20 [2.40–4.25]	< 0.0001	3.22 [2.51–4.13]	< 0.0001
Abnormal Lym	2.77 [2.40–3.18]	< 0.0001	3.93 [2.84–5.44]	< 0.0001	2.26 [1.63–3.13]	< 0.0001	5.27 [4.08–6.81]	< 0.0001
Abnormal Na	3.81 [3.22–4.49]	< 0.0001	5.26 [3.70–7.48]	< 0.0001	4.69 [3.36–6.55]	< 0.0001	4.12 [3.12–5.45]	< 0.0001
CRP > 6 mg/L	2.17 [1.85–2.56]	< 0.0001	1.76 [1.30–2.39]	0.0003	1.81 [1.37–2.40]	< 0.0001	1.83 [1.42–2.35]	< 0.0001
ESR > 10 mm/h	0.55 [0.29–1.03]	0.0613	0.90 [0.40–2.00]	0.7909	0.63 [0.35–1.13]	0.1230	0.80 [0.45–1.43]	0.4500
ALB< 35 g/L	5.57 [4.65–6.66]	< 0.0001	15.95 [9.9–25.69]	< 0.0001	11.67 [7.24–18.81]	< 0.0001	12.85 [8.93–18.50]	< 0.0001
ALT> 100 U/L	1.92 [1.51–2.45]	< 0.0001	3.44 [1.97–6.03]	< 0.0001	3.25 [1.85–5.71]	< 0.0001	8.43 [4.80–14.80]	< 0.0001
AST > 100 U/L	2.02 [1.62–2.51]	< 0.0001	5.82 [3.44–9.87]	< 0.0001	5.34 [3.20–8.89]	< 0.0001	12.66 [7.22–22.19]	< 0.0001
TBIL>17umol/L	1.61 [1.40–1.87]	< 0.0001	5.29 [2.19–12.77]	0.0002	18.34 [5.60–60.06]	< 0.0001	7.70 [4.33–13.68]	< 0.0001
SCR > 70umol/L	1.03 [0.66–1.61]	0.8935	2.48 [0.99–6.19]	0.0519	4.01 [1.60–10.04]	0.0030	5.11 [2.10–12.43]	0.0003
Proteinuria	4.94 [4.04–6.06]	< 0.0001	5.69 [4.01–8.07]	< 0.0001	3.47 [2.47–4.87]	< 0.0001	2.14 [1.64–2.78]	< 0.0001
RSV infection	1.59 [1.22–2.07]	0.0005	1.11 [0.63–1.96]	0.722	0.64 [0.30–1.36]	0.2463	0.81 [0.35–1.84]	0.6102

Definition of abnormal laboratory results: WBC, WBC count < 5.0*10^9/L or > 12*10^9/L; ALB, < 35 g/L; CRP, > 6 mg/L

*CAP* Community-acquired pneumonia, *ICU* Intensive care unit, *LBW* Low birth weight (birth weight < 2.5Kg); Unhealthy parents, at least one parent has health condition, *CHD* Congenital heart disease

**Table 5:** Univariate analysis for Risk factors of ICU admission among patients stratified by ages

Variable	< 6 m		6 m-1y		1y-2y		2y-6y	
	OR(95% CI)	***P***	OR(95% CI)	***P***	OR(95% CI)	***P***	OR(95% CI)	***P***
Gender (male)	1.09 [0.98–1.21]	0.1232	0.85 [0.67–1.09]	0.2093	0.72 [0.56–0.93]	0.0101	1.06 [0.83–1.37]	0.6203
Rural resident	1.92 [1.73–2.12]	< 0.0001	1.73 [1.37–2.17]	< 0.0001	2.11 [1.65–2.70]	< 0.0001	3.34 [2.60–4.29]	< 0.0001
Premature birth	3.38 [2.98–3.83]	< 0.0001	3.31 [2.47–4.42]	< 0.0001	2.23 [1.55–3.21]	< 0.0001	1.12 [0.69–1.82]	0.6464
LBW	3.80 [3.34–4.32]	< 0.0001	4.04 [3.05–5.35]	< 0.0001	2.15 [1.49–3.10]	< 0.0001	1.28 [0.79–2.05]	0.3141
Unhealthy parents	1.74 [1.49–2.04]	< 0.0001	1.55 [1.05–2.29]	0.0286	1.57 [1.04–2.37]	0.0308	1.58 [1.04–2.39]	0.0329
Feeding	1.43 [1.35–1.53]	< 0.0001	1.37 [1.19–1.58]	< 0.0001	1.16 [0.99–1.36]	0.0648	1.14 [0.96–1.34]	0.1292
CHD	4.66 [4.19–5.19]	< 0.0001	5.50 [4.17–7.25]	< 0.0001	4.64 [3.18–6.78]	< 0.0001	3.34 [1.91–5.83]	< 0.0001
History of pneumonia	2.53 [2.23–2.88]	< 0.0001	2.54 [1.96–3.28]	< 0.0001	2.21 [1.65–2.97]	< 0.0001	1.21 [0.85–1.73]	0.2943
History of jaundice	2.18 [1.81–2.64]	< 0.0001	3.39 [2.02–5.68]	< 0.0001	2.03 [0.84–4.91]	0.1138	1.92 [0.67–5.49]	0.2257
Cough	0.35 [0.30–0.39]	< 0.0001	0.24 [0.18–0.32]	< 0.0001	0.44 [0.32–0.60]	< 0.0001	0.30 [0.22–0.40]	< 0.0001
Fever	1.06 [0.93–1.21]	0.3493	0.92 [0.73–1.16]	0.5025	0.90 [0.69–1.16]	0.3957	0.59 [0.45–0.76]	< 0.0001
Wheezing	0.92 [0.79–1.06]	0.2301	1.14 [0.88–1.49]	0.3177	2.08 [1.55–2.79]	< 0.0001	4.48 [3.30–6.08]	< 0.0001
Cyanosis	12.46 [9.59–16.19]	< 0.0001	67.49 [19.86–229.38]	< 0.0001	29.19 [10.33–82.50]	< 0.0001	13.87 [1.95–98.85]	0.0087
Dyspnea	16.67 [9.10–30.55]	< 0.0001	57.10 [12.6–258.79]	< 0.0001	111.06 [14.18–869.53]	< 0.0001	27.99 [5.10–153.52]	< 0.0001
Abnormal chest X-ray	6.89 [5.68–8.36]	< 0.0001	12.10 [8.38–17.49]	< 0.0001	8.36 [5.63–12.42]	< 0.0001	7.39 [5.32–10.26]	< 0.0001
Abnormal WBC	2.58 [2.06–3.24]	< 0.0001	1.82 [1.17–2.85]	0.0085	1.27 [0.79–2.02]	0.3206	1.46 [0.89–2.37]	0.1322
Abnormal Neu	2.79 [2.41–3.24]	< 0.0001	3.28 [2.37–4.56]	< 0.0001	3.47 [2.52–4.78]	< 0.0001	5.25 [3.84–7.19]	< 0.0001
Abnormal Lym	3.08 [2.65–3.59]	< 0.0001	3.67 [2.58–5.21]	< 0.0001	2.75 [1.93–3.92]	< 0.0001	7.25 [5.32–9.88]	< 0.0001
Abnormal Na	4.00 [3.38–4.75]	< 0.0001	5.40 [3.72–7.84]	< 0.0001	5.94 [4.15–8.49]	< 0.0001	4.99 [3.62–6.87]	< 0.0001
CRP > 6 mg/L	2.18 [1.84–2.57]	< 0.0001	1.87 [1.35–2.58]	0.0001	1.80 [1.32–2.46]	0.0002	1.94 [1.44–2.62]	< 0.0001
ESR > 10 mm/h	0.55 [0.29–1.06]	0.0737	1.13 [0.44–2.90]	0.7952	0.47 [0.24–0.89]	0.0212	0.53 [0.27–1.04]	0.0657
ALB< 35 g/L	5.98 [4.99–7.16]	< 0.0001	17.47 [10.81–28.22]	< 0.0001	11.31 [6.97–18.34]	0.0028	12.46 [8.5–18.28]	< 0.0001
ALT> 100 U/L	2.06 [1.60–2.64]	< 0.0001	4.33 [2.46–7.61]	< 0.0001	2.60 [1.36–4.97]	< 0.0001	14.47 [8.25–25.37]	< 0.0001
AST > 100 U/L	1.99 [1.59–2.50]	< 0.0001	5.88 [3.40–10.16]	< 0.0001	5.31 [3.09–9.13]	< 0.0001	9.98 [5.53–18.02]	< 0.0001
TBIL>17umol/L	1.69 [1.45–1.97]	< 0.0001	5.33 [2.14–13.26]	< 0.0001	13.04 [4.33–39.26]	< 0.0001	12.46 [8.50–18.28]	< 0.0001
SCR > 70umol/L	1.20 [0.77–1.89]	0.4203	3.07 [1.23–7.68]	0.0166	3.43 [1.24–9.47]	0.0176	8.35 [3.42–20.41]	< 0.0001
Proteinuria	4.96 [4.05–6.08]	< 0.0001	5.95 [4.14–8.57]	< 0.0001	3.57 [2.47–5.16]	< 0.0001	2.41 [1.77–3.29]	< 0.0001
RSV infection	1.29 [0.98–1.69]	0.0644	1.28 [0.71–2.32]	0.4109	0.61 [0.26–1.42]	0.2494	0.41 [0.12–1.36]	0.1449

*CAP* Community-acquired pneumonia, *ICU* Intensive care unit, *LBW* Low birth weight (birth weight < 2.5Kg); Unhealthy parents, at least one parent has health condition, *CHD* Congenital heart disease, *WBC* White blood cell, *Neu* Neutrophil count, *Lym* Lymphocyte count, *Na* Serum sodium, *CRP* C-reactive protein, *ESR* Erythrocyte sedimentation rate, *ALB* Albumin, *AST* Aspartate aminotransferase, *ALT* Alanine aminotransferase (ALT), *TBIL* Total bilirubin (TBIL), *SCR* Serum creatine, *RSV* Respiratory syncytial virus

### Independent risk factors for severe CAP identified by multivariate analysis

All the potential risk factors identified through the univariate analysis of overall patients or subgroup patients were applied to multivariate analysis to decide the independent risk factors for severe CAP or ICU admission during hospitalization (Fig. 1). When it comes to all the patients enrolled, factors that independently predicted an increased risk of severe CAP included rural residency, low birth weight, CHD as an underlying medical condition, symptoms on admission like wheezing and cyanosis, abnormal chest X-ray, abnormal neutrophils count, abnormal lymphocytes count, abnormal serum sodium level, hypoalbuminemia (ALB < 35 g/L), proteinuria, and RSV infection. Independent risk factors for ICU admission among overall patients were similar to that of severe CAP, except that premature birth, patients with health issues, history of pneumonia, dyspnea or no fever at admission, and elevated CRP (CRP > 6 mg/L) were also added to the list of independent predictors.

Independent risk factors identified for subgroup patients of different age range differed from one another. As for the infant patients born within 6 months, independent risk factors for developing severe CAP or requiring ICU referral included rural residency, premature or LBW, formula feeding, having CHD, cyanosis on admission, abnormal X-ray, abnormal neutrophils count, abnormal lymphocytes count, abnormal serum sodium level, hypoalbuminemia (ALB < 35 g/L), proteinuria, and RSV infection (*P* <  0.05).

Independent risk factors for developing severe CAP or requiring ICU referral among children aged between 6 months and 1 year included history CHD, abnormal X-ray, and abnormal lymphocytes count. Rural residency and proteinuria were also independent risk factors for ICU admission children aged between 6 months and 1 year. For children aged between 1 year and 2 years, hypoalbuminemia (ALB < 35 g/L) was the only independent risk factor associated with an increased risk of developing severe CAP, while low birth weight and proteinuria were the only two independent risk factors for ICU admission. When it comes to children aged between 2 years and 6 years, independent risk factors for severe CAP included wheezing on admission, abnormal neutrophils count, abnormal lymphocytes count, abnormal serum sodium level, and hypoalbuminemia (ALB < 35 g/L). Risk factors associated with ICU admission for children of 2–6 years old were similar to that of developing severe CAP, except for having rural residency and cough at admission also added to the list. Specifically, wheezing was an independent predictor of developing severe CAP or requiring ICU referral for the older children (2–6 years old) but not the pediatric patients of younger age groups.

## Discussion

Severe community-acquired pneumonia (CAP) remains the leading cause of pediatric mortality [1]. Identifying risk factors associated with the development of severe CAP among pediatric CAP patients, especially for children of different age groups, is useful in guiding the clinical practice and improving outcomes. Utilizing the abundant clinical data in our center, we retrospectively identified risk factors associated with severe CAP or ICU admission among children hospitalized with CAP, which included rural residency, premature birth, low birth weight, patients with health issues, formula feeding, preexisted congenital heart disease, history of pneumonia, wheezing, cyanosis, abnormal chest X-ray, abnormal WBC, hypoalbuminemia, elevated CRP, proteinuria, and RSV infection. Subgroup analysis according to age stratification revealed that risk factors might vary with age. Birth history, feeding history, history of pneumonia, cyanosis or dyspnea on admission, and RSV infection only demonstrated a significant impact on younger children (< 1 year old). As a presenting feature, wheezing was a significant risk factor for severe CAP among older children (2–6 years old) with CAP but not among the younger ones. Our study systematically demonstrated the age-specific risk factors for developing the severe disease among children hospitalized with CAP, adding to the evidence of risk stratification of pediatric CAP and better guiding the clinical practice.

As shown in our study, infant patients born within 6 months constituted a major component of pediatric patients hospitalized for CAP, accounting for nearly half the population of the overall patients aged less than 6 years old. Patients of different age groups differed significantly from one another in terms of clinical manifestations, radiographic findings, medical history, and, most importantly, disease severity. Younger age was shown to be a significant predictor of severe CAP in the present study, which was consistent with the previous studies [8, 10, 12]. Specifically, we found that infants born within 6 months demonstrated a significantly higher risk of developing severe CAP than older children. It could be attributed to the underdeveloped immune system and the immature respiratory system that predisposes to developing the severe disease among infants [18]. Besides, the symptoms in the infants could be more insidious and undetectable at the very early stage, in which case appropriate treatment is delayed, thus leading to the development of severe disease [19].

In previous studies, birth history was either not included in the analysis of risk factors or turned out to be irrelevant to disease severity [8]. In the present study, premature birth and low birth weight were found to be significantly associated with an increased risk of developing CAP among pediatric patients hospitalized with CAP. According to age stratification, further subgroup analysis revealed that birth history was a significant predictor only among younger patients, especially for infants younger than 6 months. Our findings indicated that the impact of birth history on disease severity of CAP would decrease as the children grow up. These findings were readily understandable, as the compromised health status caused by premature birth or low birth weight would only last for a couple of months and would soon recover to normal as the children grow up [20]. The different conclusions regarding the significance of birth history in the previous studies could be attributed to the different composition of study subjects.

Like birth history, feeding history (formula feeding) predicted an increased risk of developing severe CAP only among infants born within 6 months. It is well-known that breastfeeding within the first 6 months of birth can better preserve infants’ immunity by supplementing the immunoglobulin that infants cannot produce themselves yet [21, 22]. Infants fed with formula substances have a weaker immune response in the fight against pulmonary infection [22], thereby predisposing to the development of severe infection. As the immune system gets mature, the feeding method loses its significant impact on children’s immunity. Our findings highlight the importance of breastfeeding within the first 6 months after birth.

Medical history of congenital heart disease (CHD) and pneumonia also increases the risk of severe CAP; however, the impact differs across different age groups. CHD is a significant predictor of severity among pediatric patients younger than 2 years. Mostly, CHD is fixed by surgical treatment during the first 2 years of life [23, 24], making CHD history irrelevant to disease severity among children aged between 2 and 6 years. On the contrary, a history of pneumonia is associated with an increased incidence of severe CAP only among children older than 6 months. Previous pneumonia episodes usually leave behind residues of structural damage or fibrosis lesion [25], which predisposes to the development of severe disease in the new round of pneumonia.

As proven by our study, symptoms demonstrated at admission convey clinical value in predicting disease severity. For example, cyanosis was a significant predictor of severe CAP across patients of different age groups. Cyanosis, as a sign of systematic hypoxia, would occur only when ventilation function was severely compromised [26, 27]. Instead of being considered a predictor, cyanosis is actually the sign of severe disease and usually associated with the development of severe complications, such as acute respiratory distress syndrome, pulmonary congestion, hydrothorax, etc. [27]. As a sign of airway hyperresponsiveness, wheezing is a common symptom in pediatric CAP and is usually associated with viral infection [28–30]. Here in our study, wheezing was found to be a significant risk factor for severe CAP. Interestingly, the predicting value of wheezing is most notable among older children (aged between 2 to 6 years) but less significant or not significant among younger kids. It could be attributed to the difficulty in detecting wheezing among infants. One of the unexpected findings is that patients who manifested no fever on admission had a higher risk of requiring ICU referral. One possible explanation is that the severity of CAP without fever on admission is easily neglected, thus delaying the appropriate treatment. In comparison, cases with fever tend to be treated more intensively, which effectively prevents disease progression.

Chest radiography is the routine test in the diagnosis of CAP [31]. Although patients with CAP may not necessarily demonstrate abnormalities on radiography [31], previous studies have shown that radiologic evidence of pneumonia can predict progressive disease among children with CAP. Consistently, our study also found that abnormalities on radiographs, including signs of pneumonia, pulmonary consolidation, pneumothorax, hydrothorax, etc., are significantly correlated with increased risk of developing severe CAP or requiring ICU referral across pediatric CAP patients of different age groups. Chest radiography seems to be an even more important predictor among younger patients, as shown in our results.

Biomarkers like white blood cell (WBC) count and serum C-reactive protein (CRP) concentration are commonly used to diagnose CAP and define its etiology [32]. An elevation in CRP levels is generally considered as proof of bacterial infection [33]. WBC’s application in identifying bacterial and nonbacterial pediatric CAP has been proved to be less reliable by more and more recent studies [34, 35]. Yet both of them could be a useful predictor of disease severity in pediatric CAP, as shown in a previous report [8]. Our study also confirmed that abnormal WBC count or elevated CRP levels were significantly associated with an increased risk of severe CAP, especially among younger patients. As an indicator of nutritional status, serum albumin has been associated with the risk of progressive disease among patients with pneumonia [36–38]. Similarly, our study also found that reduced albumin levels (albumin < 30 g/L) are a significant predictor of developing severe CAP or requiring ICU referral among children of different age groups. Hypoalbuminemia is usually associated with compromised immune status due to malnutrition [39]. Moreover, hypoalbuminemia (albumin < 30 g/L) could reduce plasma osmolality and enhance the exudation within the lungs, thus, exacerbating the infection [40]. Therefore, it explains the involvement of albumin in the disease severity of CAP. Renal involvement, such as acute glomerulonephritis, is a common complication of pneumonia, especially in severe cases [41, 42]. Proteinuria, as an indicator of glomerulonephritis, is found to run a high incidence among patients hospitalized with pneumonia and is an independent risk factor for extended hospital stay and admission to ICU [43]. Our study also confirmed proteinuria as an independent risk factor for severe CAP among children of all age groups with CAP.

Respiratory syncytial virus (RSV) is one of the most common causative pathogens of pediatric CAP among the Chinese population [44]. RSV has been reported to be the most frequent viral pathogen in children with severe CAP [45, 46]. In our study, RSV was detected in up to 20% of the children hospitalized with CAP and was identified as an independent risk factor for the severe disease among infants born within 6 months.

It is worth mentioning that only one independent risk factor (hypoalbuminemia) was detected for children aged between 1 and 2 years. It suggests that less reliable factors could be applied in predicting the risk of severe CAP or ICU admission among children of this age group. The negative findings for most other factors could be attributed to the modest sample size of this specific age group.

Our study is the first to investigate the age-specific risk factors for severe disease among children hospitalized with CAP. Although some limitations of the present study also need to be addressed. Firstly, we did not evaluate risk factors associated with dismal outcomes, such as death, in our analysis because of the low incidence of mortality of the study population. Also, long-term outcomes, such as disease recurrence, were not analyzed due to the lack of pertinent data. Secondly, as a retrospective study, some information was missed out or unavailable for some patients. To guarantee the data acquisition for most patients, our analysis only included the routine items in the medical records. Last but not least, clinical management was not evaluated in the current study, and thus we could not make any conclusions regarding the relationship between therapeutic strategy and disease progression.

In conclusion, our study proves that risk factors associated with developing the severe disease among children hospitalized with CAP vary with age. An age-specific model should be developed for risk stratification of pediatric CAP patients, which could better guide the practice of precision medicine.

## Data Availability

All the data applied in the present were derived from the medical system of our hospital and was approved by the hospital research committee.

## Abbreviations

ALB: Albumin
CAP: Community-acquired pneumonia
CHD: Congenital heart disease
CRP: C-reactive protein
LBW: Low birth weight
RSV: Respiratory syncytial virus
WBC: White blood cell
WHO: World Health Organization
